# *Streptococcus agalactiae* in Brazil: serotype distribution, virulence determinants and antimicrobial susceptibility

**DOI:** 10.1186/1471-2334-14-323

**Published:** 2014-06-12

**Authors:** Vanusa G Dutra, Valéria MN Alves, André N Olendzki, Cicero AG Dias, Alessandra FA de Bastos, Gianni O Santos, Efigênia LT de Amorin, Meireille ÂB Sousa, Rosemary Santos, Patricia CS Ribeiro, Cleuber F Fontes, Marco Andrey, Kedma Magalhães, Ana A Araujo, Lilian F Paffadore, Camila Marconi, Eddie FC Murta, Paulo C Fernandes Jr, Maria SG Raddi, Penélope S Marinho, Rita BG Bornia, Jussara K Palmeiro, Libera M Dalla-Costa, Tatiana CA Pinto, Ana Caroline N Botelho, Lúcia M Teixeira, Sérgio Eduardo L Fracalanzza

**Affiliations:** 1 Instituto de Biologia do Exército, Rio de Janeiro, Brazil; 2 Universidade Estadual de Campinas, Campinas, São Paulo, Brazil; 3 Hospital Naval Marcílio Dias, Rio de Janeiro, Brazil; 4 Fundação Faculdade Federal de Ciências Médicas, and Laboratório Hospital Mãe de Deus, Porto Alegre, Rio Grande do Sul, Brazil; 5 Laboratório Sabin, Brasília, Distrito Federal, Brazil; 6 Laboratório Sérgio Franco, Rio de Janeiro, Brazil; 7 Laboratório Hermes Pardini, Belo Horizonte, Minas Gerais, Brazil; 8 Hospital da Universidade Federal do Amazonas, Manaus, Amazonas, Brazil; 9 Hospital da Universidade Federal do Maranhão, São Luis, Maranhão, Brazil; 10 Universidade Federal da Bahia, Salvador, Bahia, Brazil; 11 Hospital da Universidade de Cuiabá, Cuiabá, Mato Grosso, Brazil; 12 Laboratório Municipal de Saúde Pública de Recife, Recife, Pernambuco, Brazil; 13 Hospital da Universidade de São Paulo, São Paulo, Brazil; 14 Universidade Estadual Paulista, Campus Botucatu, São Paulo, Brazil; 15 Universidade Federal do Triângulo Mineiro, Uberaba, Minas Gerais, Brazil; 16 Universidade Estadual Paulista, Campus Araraquara, São Paulo, Brazil; 17 Maternidade Escola, Universidade Federal do Rio de Janeiro, Rio de Janeiro, Brazil; 18 Laboratório de Bacteriologia, Hospital de Clínicas, Universidade Federal do Paraná, Curitiba, Paraná, Brazil; 19 Departamento de Microbiologia Médica, Instituto de Microbiologia Paulo de Góes, Universidade Federal do Rio de Janeiro, Rio de Janeiro, Brazil

## Abstract

**Background:**

Group B *Streptococcus* (GBS) remains a major cause of neonatal sepsis and is also associated with invasive and noninvasive infections in pregnant women and non-pregnant adults, elderly and patients with underlying medical conditions. Ten capsular serotypes have been recognized, and determination of their distribution within a specific population or geographical region is important as they are major targets for the development of vaccine strategies. We have evaluated the characteristics of GBS isolates recovered from individuals with infections or colonization by this microorganism, living in different geographic regions of Brazil.

**Methods:**

A total of 434 isolates were identified and serotyped by conventional phenotypic tests. The determination of antimicrobial susceptibility was performed by the disk diffusion method. Genes associated with resistance to erythromycin (*erm*A, *erm*B, *mef*A) and tetracycline (*tet*K, *tet*L, *tet*M, *tet*O) as well as virulence-associated genes (*bac, bca, lmb, scp*B) were investigated using PCR. Pulsed-field gel electrophoresis (PFGE) was used to examine the genetic diversity of macrolide-resistant and of a number of selected macrolide-susceptible isolates.

**Results:**

Overall, serotypes Ia (27.6%), II (19.1%), Ib (18.7%) and V (13.6%) were the most predominant, followed by serotypes IV (8.1%) and III (6.7%). All the isolates were susceptible to the beta-lactam antimicrobials tested and 97% were resistant to tetracycline. Resistance to erythromycin and clindamycin were found in 4.1% and 3% of the isolates, respectively. Among the resistance genes investigated, *tet*M (99.3%) and *tet*O (1.8%) were detected among tetracycline-resistant isolates and *erm*A (39%) and *erm*B (27.6%) were found among macrolide-resistant isolates. The *lmb* and *scp*B virulence genes were detected in all isolates, while *bac* and *bca* were detected in 57 (13.1%) and 237 (54.6%) isolates, respectively. Molecular typing by PFGE showed that resistance to erythromycin was associated with a variety of clones.

**Conclusion:**

These findings indicate that GBS isolates circulating in Brazil have a variety of phenotypic and genotypic characteristics, and suggest that macrolide-resistant isolates may arise by both clonal spread and independent acquisition of resistance genes.

## Background

*Streptococcus agalactiae* (Group B *Streptococcus*, GBS) is a leading cause of neonatal morbidity and mortality [1], and is also an important opportunistic agent of infections in pregnant women, as well as non-pregnant adults, especially the elderly or those with underlying medical conditions [2]. This microorganism is commonly found as a colonizer of the genital and the gastrointestinal tracts of both men and women, and vertical transmission from a colonized mother to her newborn during labor can result in life threatening infections.

GBS virulence is complex and multifactorial. Several virulence determinants are involved in the adhesion to and invasion of host cells, as well as in the immune system evasion. Surface components, including a polysaccharide capsule and proteins, such as Cα, Cβ, Rib and the laminin binding protein (LMB), and a number of enzymes (like the C5a peptidase) and toxins/cytolysins, are produced and have been associated with GBS virulence [3].

Capsular polysaccharides are recognized as playing a key role as virulence factors and are important targets for the development of vaccine strategies. GBS capsular polysaccharides have chemical and antigenic differences that enable the subdivision of this species into ten serotypes, denominated Ia, Ib, II-IX [4]. Vaccines against GBS infections must include the more common serotypes associated with disease in different populations and capsular polysaccharide-protein conjugate vaccines are in clinical trials [5]. The epidemiological distribution of these serotypes can vary according to several aspects, including the geographical region, the profile of the population being studied, and the source of the bacterial isolate [6]. Four serotypes (Ia, II, III and V) are usually the most frequently isolated in the United States and in some European countries [6-8], while serotypes VI to IX are rarely described [4,9]. In Brazil, the occurrence of serotypes Ia, Ib, II, III, IV and V has been described in a few studies conducted with isolates originated from the South and the Southeast regions [10-15].

*S. agalactiae* is still uniformly sensitive to penicillin, although isolates with reduced susceptibility to penicillin have been reported since 2008 [16-18]. Clindamycin or erythromycin is recommended for GBS intrapartum prophylaxis for penicillin-allergic women with high risk of anaphylaxis or when therapeutic failure is suspected [19]. However, increasing rates of resistance to these antibiotics have been detected in several regions of the world, including Europe [8,20], Asia [21], North America [22-24], and South America [11,13,15,25].

Despite the clinical and epidemiological impact of GBS infections and the trends to increasing occurrence of antimicrobial resistance, information about isolates from Brazil is still fragmentary and usually restricted to a few areas of this large country. There is no Brazilian study that provides a national overview of the phenotypic and genotypic characteristics of circulating isolates of GBS. In the present report we describe the serotype distribution, antimicrobial susceptibility, presence of virulence-related and antimicrobial resistance genes among GBS isolates collected from the five geographical regions in Brazil. Also, the genetic diversity of macrolide-resistant isolates was assessed and compared to a representative fraction of the macrolide-susceptible isolates, providing a national overview of the characteristics of strains circulating in the country.

## Methods

### Bacteria

Four hundred thirty four *S. agalactiae* isolates were included in this study. They were obtained during March 2005 to December 2009 from patients living in the five Brazilian geographical regions: South region [Paraná (35 isolates), Rio Grande do Sul (59 isolates)]; Southeast region [São Paulo (100 isolates), Rio de Janeiro (92 isolates), Minas Gerais (18 isolates)]; Mid-West region [Mato Grosso (16 isolates); Brasília (25 isolates)]; Northeast region [Bahia (17 isolates), Maranhão (27 isolates), Pernambuco (40 isolates)]; and North region [Amazonas (five isolates)]. The sources of the isolates included two groups of specimens: (i) from colonized patients [vaginal and perianal secretions (249 isolates)], (ii) from symptomatic adults [Table 1 (185 isolates)].

**Table 1:** **Serotype distribution of the 185 *Streptococcus agalactiae *** **isolates recovered from symptomatic adults included in the present study, according to the type of clinical specimen and geographical origin**

**Clinical specimen (number of isolates)**	**Geographical region (number of isolates)**	**Serotype (number of isolates)**^**b**^
Urine (167)	South (83)	Ia (35); Ib (11); II (12); III (6); IV (7); V (9); NT (3)
	Southeast (57)	Ia (13); Ib (10); II (9); III (6); IV (6); V (6); NT (7)
	Mid-west (27)	Ia (12); Ib (2); II (3); III (2); IV (3); V (2); NT (3)
Blood and other sterile fluids (10)	Southeast (10)	Ia (3); Ib (3); II (3); IV (1)
Male genital tract discharges (3)	Mid-west (2)	Ia (1); II (1)
	Southeast (1)	II (1)
Other^a^ (5)	Southeast (5)	Ia (1); Ib (3); II (1)
Total (185)	All above (185)	Ia (65); Ib (29); II (30); III (14); IV (17); V (17); NT (13)

^a^Other clinical specimens included abscesses and ear secretions.

^b^NT, nontypeable by using antisera against Ia, Ib, II-VIII serotypes.

Conventional methods were used for culturing procedures and identification of the isolates as GBS [19]. Serological grouping was performed using a commercial latex agglutination test, according to the manufacturer's instructions (Slidex Strepto Kit, bioMerieux, France). All of the strains were stored in Todd-Hewitt broth (Difco Laboratories, Detroit, Michigan, USA) with glycerol at -20°C.

The project was approved by the Research Ethics Committee of the Hospital Universitário of Universidade Federal do Rio de Janeiro (protocol number 21905), and written informed consents were obtained from all participants of the study.

### Serotyping

All the isolates were serotyped by using the HCl extraction method and double immunodiffusion tests or capillary precipitation tests with typing antisera against nine of the capsular polysaccharides (Ia, Ib and II-VIII), prepared in house according to standardized methods [10]. Serotype IX was not investigated. Nontypeable isolates were designated as NT.

### Antimicrobial susceptibility testing

All the 434 isolates were tested for ampicillin, cefotaxime, clindamycin, chloramphenicol, erythromycin, levofloxacin, and tetracycline (Oxoid) susceptibility by disk diffusion according to the CLSI guidelines [26]. The erythromycin-clindamycin double-disk test was used to determine the resistance phenotypes.

### Detection of antimicrobial resistance genes

Macrolide and/or tetracycline-resistant isolates, as defined by phenotypic methods, were tested for the presence of the *erm*A, *erm*B, *mef*A, *tet*K, *tet*L, *tet*M and *tet*O genes by PCR using previously described primers [27,28].

### Detection of virulence-associated genes

The presence of the *bac, bca, lmb* and *scpB* genes, which encode for alpha and beta proteins, laminin binding protein, and C5a peptidase, respectively, was evaluated in all 434 isolates by PCR using previously described primers [29,30].

### Molecular typing by PFGE

All the erythromycin-resistant isolates (total of 18 strains) and 25 randomly selected erythromycin-susceptible isolates were characterized by PFGE. Chromosomal DNA was prepared in agarose plugs as previously described [31] and treated with 12U of *Sma*I (Invitrogen, San Diego, CA) for 18–24 h at 25°C. The fragments were separated by PFGE in 1.2% agarose gels in a CHEF-DR III system (Bio-Rad Laboratories, Hercules, CA) with pulse times of 2 to 30 s for 23 h at 11.3°C and 6 V/cm. The restriction profiles were analyzed by using the BioNumerics software version 6.6 (Applied Maths, Ghent, East Flanders, Belgium). The Dice similarity coefficient was used to determine the similarity between each banding profiles, and a dendrogram was constructed using the unweighted-pair group method with arithmetic averages (UPGMA) with a tolerance coefficient of 1.0%. Isolates with similarities of ≥ 70% were considered as closely related and were clustered in a given clonal complex (CC). The clonal complexes (CCs) were assigned with capital letters (A to I). Isolates with similarities of < 70% were considered genetically unrelated.

### Statistical analysis

The statistical analyses were performed using the SPSS program (“Statistical Package for the Social Sciences” version 19.0, IBM Brazil, São Paulo, SP, Brazil). The chi-square test was used to examine differences in serotype distribution between isolates recovered from infection and colonization specimens, as well as to evaluate correlation between erythromycin-resistance phenotypes and genotypes. Good evidence against the null hypothesis was considered with *p* values of <0.05.

The genetic diversity, as revealed by using PFGE, was calculated using the Simpson’s Index of Diversity (SID). The 95% confidence intervals (95% CI) were also calculated [32,33].

## Results

Overall, serotypes Ia (120 isolates; 27.6% ± 4.2, 95% CI), II (83 isolates; 19.1% ± 3.7), Ib (81 isolates; 18.7% ± 3.7) and V (59 isolates; 13.6% ± 3.2) were the most predominant among the 434 GBS isolates included in the present study, followed by serotypes IV (35 isolates; 8.1% ± 2.6) and III (29 isolates; 6.7% ± 2.3). The sum of the four most common serotypes (Ia, Ib, II and V) constituted 79% (±3.8) of the isolates while percentages above 90% were obtained with the addition of serotypes III and IV. Serotypes VI, VII, and VIII were not found and 6.2% ± 2.3 (27 isolates) of the isolates were nontypeable.

**Figure 1:**
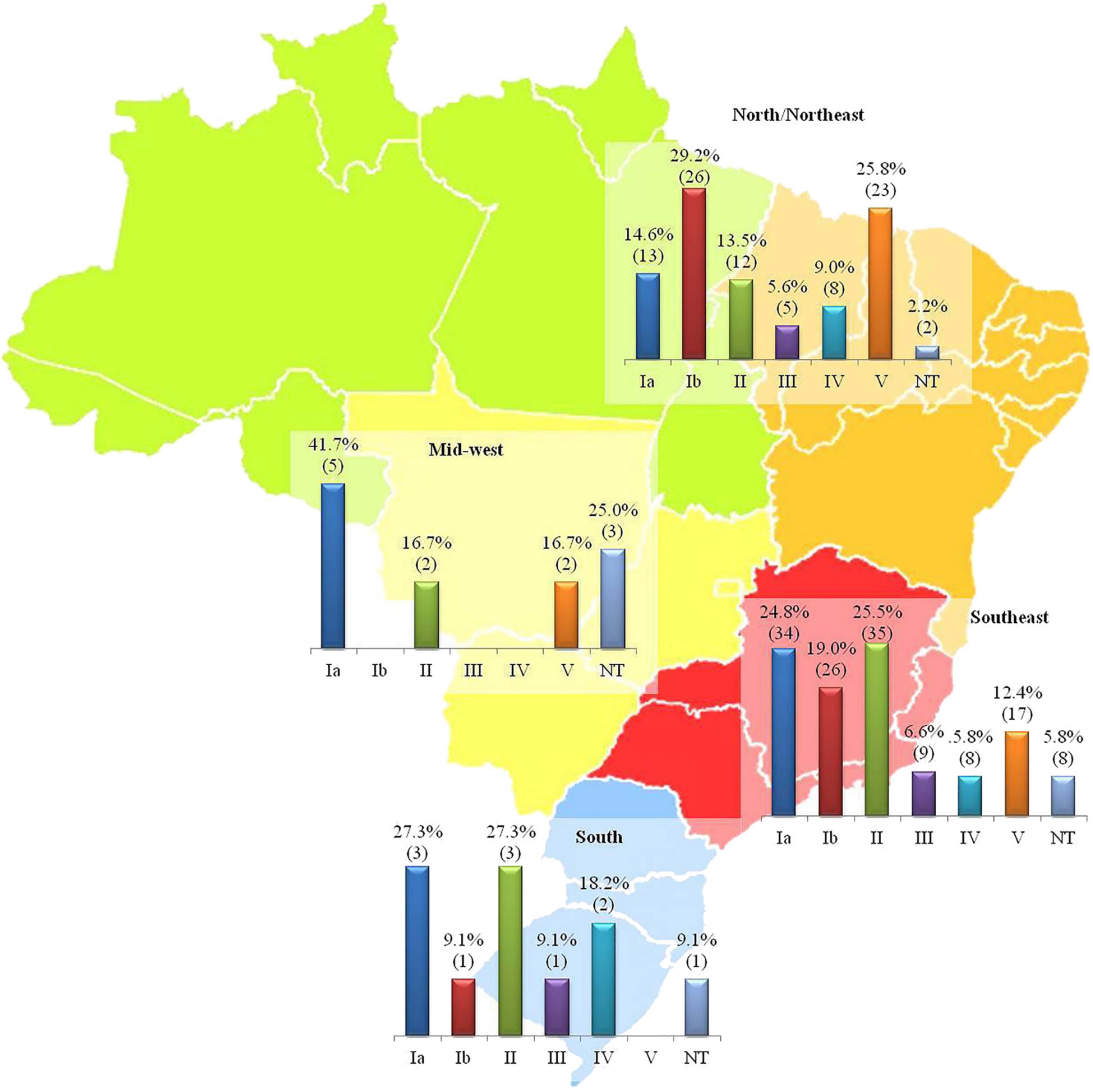
**Serotype distribution among the 249 *Streptococcus agalactiae *isolates recovered from colonization included in the present study, according to geographical region.** NT, nontypeable by using antisera against Ia, Ib, II-VIII serotypes.

Serotypes Ia, II and Ib were the most frequent among isolates recovered from infections, which were represented by the South, Southeast and Mid-West regions (Table 1). Although absolute numbers of colonization isolates were small in some regions, particularly the Mid-West, serotypes Ia and II were the most frequently observed in the South, Southeast and Mid-West regions, whereas serotypes Ib and V were the most common in the North/Northeast region (Figure 1).

Tests for detection of the virulence genes showed that all the 434 GBS isolates harbored both the *lmb* and *scp*B genes, whereas the *bca* and *bac* genes were detected in 54.4% ± 4.7 (236 isolates) and 13.1% ± 3.2 (57 isolates) of the isolates, respectively. The majority of isolates from infections (101 isolates; 54.6% ± 4.7) had neither *bac* or *bca* genes, while almost half of the colonization strains (122 isolates; 49% ± 4.7) were *bca*-positive (Table 2).

**Table 2:** **Distribution of *bac *** **and *bca *** **virulence-associated genes according to serotype and clinical origin of the 434 *Streptococcus agalactiae *** **isolates included in the present study**

**Serotype (number of isolates)**	**Clinical origin (number of isolates)**	**Number (%) of *bac *** **(+) isolates**	**Number (%) of *bca *** **(+) isolates**	**Number (%) of *bac *** **(+)/*bca *** **(+) isolates**	**Number (%) of *bac *** **(-)/*bca *** **(-) isolates**
Ia (120)	Colonization (55)	1 (1.8%)	31 (56.4%)	6 (10.9%)	17 (30.9%)
	Infection (65)	2 (3.1%)	26 (40%)	3 (4.6%)	34 (52.3%)
Ib (81)	Colonization (53)	1 (1.9%)	23 (43.4%)	10 (18.9%)	19 (35.8%)
	Infection (28)	0	10 (35.7%)	8 (28.6%)	10 (35.7%)
II (83)	Colonization (52)	2 (3.8%)	26 (50%)	8 (15.4%)	16 (30.8%)
	Infection (31)	0	12 (38.7%)	2 (6.5%)	17 (54.8%)
III (29)	Colonization (15)	2 (13.3%)	7 (46.7%)	0	6 (40%)
	Infection (14)	0	3 (21.4%)	0	11 (78.6%)
IV (35)	Colonization (18)	0	11 (61.1%)	2 (11.1%)	5 (27.8%)
	Infection (17)	0	9 (52.9%)	1 (5.9%)	7 (41.2%)
V (59)	Colonization (42)	1 (2.4%)	18 (42.8%)	2 (4.8%)	21 (50%)
	Infection (17)	0	3 (17.6%)	1 (5.9%)	13 (76.5%)
NT^a^ (27)	Colonization (14)	1 (7.1%)	6 (42.9%)	4 (28.6%)	3 (21.4%)
	Infection (13)	0	4 (30.8%)	0	9 (69.2%)
Total (434)	Colonization (249)	8 (3.2%)	122 (49%)	32 (12.9%)	87 (34.9%)
	Infection (185)	2 (1.1%)	67 (36.2%)	15 (8.1%)	101 (54.6%)

^a^NT, nontypeable by using antisera against Ia, Ib, II-VIII serotypes.

All the 434 isolates, regardless serotype, were positive for the *scp*B and *lmb* virulence-associated genes.

Uniform susceptibility was detected to ampicillin, levofloxacin, cefotaxime, and chloramphenicol, while most (97% ± 1.6) of the isolates were resistant to tetracycline. Resistance to erythromycin and clindamycin was found in 4.1% ± 1.8 (18 isolates) and 3% ± 1.6 (13 isolates) of the isolates, respectively. Among the erythromycin-resistant (Ery^R^) isolates, the constitutive macrolide-lincosamide-streptogramin B (cMLS_B_) phenotype was the predominant phenotype (13/18 isolates) followed by the M (3/18 isolates) and the inducible MLS_B_ (2/18 isolates) phenotypes. The relationship among resistance phenotypes and genotypes was significant (*p* = 0.028). All the isolates with iMLS_B_ and M phenotypes and 2 of the 13 isolates with cMLS_B_ phenotype harbored the *erm*A gene, whereas the *erm*B gene was detected in five isolates expressing the cMLS_B_ phenotype. Six isolates belonging to the cMLS_B_ phenotype did not show the presence of the *erm* or *mef* genes, even after several attempts. Erythromycin-resistant strains were represented by multiple serotypes (Table 3).

**Table 3:** **Distribution of phenotypic and genotypic characteristics among the 18 macrolide-resistant *Streptococcus agalactiae *** **isolates included in the present study**

**Isolate**	**City of origin**^**a**^	**Serotype**	**Phenotype**^**b**^	**Genotype**^**c**^		
				***erm*** **A**	***erm*** **B**	***mef*** **A**
4676	Rio de Janeiro	Ia	iMLS_B_	+	-	-
4677	Rio de Janeiro	Ia	M	+	-	-
4583	Rio de Janeiro	NT^d^	cMLS_B_	-	+	-
4740	Rio de Janeiro	III	cMLS_B_	-	-	-
4714	Rio de Janeiro	II	cMLS_B_	-	-	-
4835	Rio de Janeiro	II	M	+	-	-
5008	Rio de Janeiro	III	cMLS_B_	-	+	-
4619	Rio de Janeiro	NT^d^	cMLS_B_	-	-	-
282	Rio de Janeiro	II	cMLS_B_	-	-	-
4971	Rio de Janeiro	Ia	cMLS_B_	-	+	-
907	Rio de Janeiro	II	cMLS_B_	-	-	-
1196	Rio de Janeiro	Ib	cMLS_B_	-	-	-
4501	Porto Alegre	III	M	+	-	-
4555	Porto Alegre	Ia	cMLS_B_	+	-	-
4732	Brasília	Ia	cMLS_B_	-	+	-
4736	Brasília	Ia	cMLS_B_	-	+	-
6717	Recife	V	iMLS_B_	+	-	-
8134	Manaus	Ib	cMLS_B_	+	-	-

^a^Rio de Janeiro is located in the Southeast region; Porto Alegre is located in the South region; Brasilia is located in the Mid-West; Recife is located in the Northeast and Manaus is located in the North region.

^b^cMLS_B_: constitutive macrolide-lincosamide-streptogramin B resistance phenotype; iMLS_B_: inducible macrolide-lincosamide-streptogramin B resistance phenotype; M: macrolide resistance phenotype.

^c^*erm*: erythromycin ribosome methylation; *mef*: macrolide efflux.

^d^NT, nontypeable by using antisera against Ia, Ib, II-VIII serotypes.

Two tetracycline resistance genes, *tet*M and *tet*O, were detected in 99.3% ± 0.8 and 1.8% ± 1.2 of the total GBS isolates, respectively, and five of them harbored both genes. The *tet*K and *tet*L genes were not detected.

**Figure 2:**
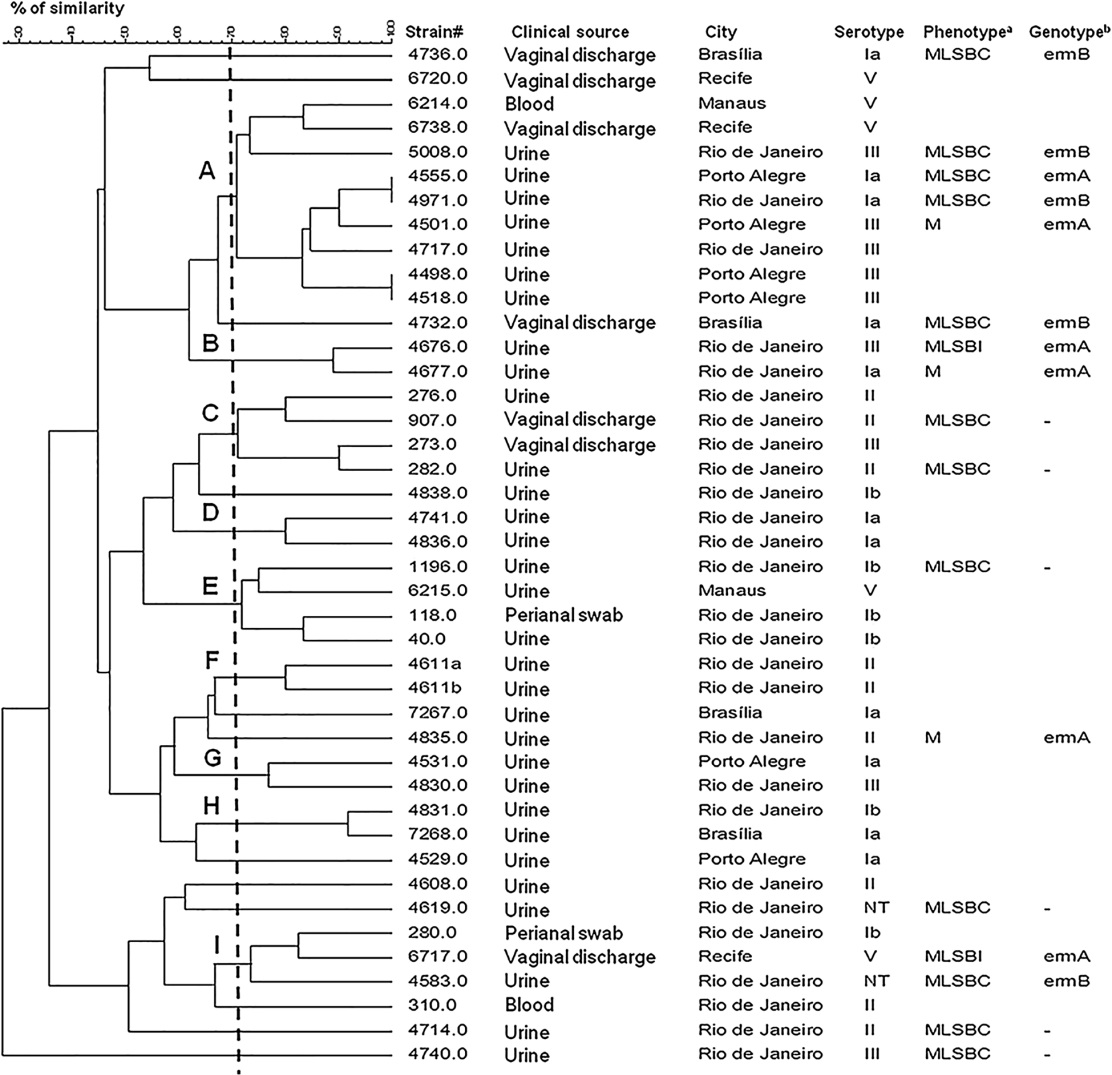
**Dendrogram constructed by similarity and clustering analysis using the Dice coefficient and UPGMA of the digitalized PFGE profiles of 42 *Streptococcus agalactiae *isolates included in the present study.** A total of 17 erythromycin-resistant isolates and 25 erythromycin-susceptible isolates were included. A tolerance of 1% was applied. The vertical line indicates the 70% level of similarity. The upper-case letters indicate the clonal complexes. cMLS_B_: constitutive resistance to macrolide-lincosamide-streptogramin B; iMLS_B_: inducible resistance to macrolide-lincosamide-streptogramin B; M: resistance to erythromycin only. (-): indicates the absence of resistance-associated genes.

One Ery^R^ strain could not be typed by PFGE, despite several attempts, due to resistance to *Sma*I digestion. Among the remaining 17 isolates, 5 CCs and 16 different PFGE profiles were identified, and the Simpson’s Index of Diversity (SID) for this subgroup of isolates was 0.897 (95% CI, 0.807-0.987). Among the 25 erythromycin-susceptible strains, 9 CCs and 25 different profiles were observed, with a SID of 0.960 (95% CI, 0.940-0.980). Overall, 9 CCs and 40 different PFGE profiles were identified among the 42 isolates (Figure 2), with a SID of 0.935 (95% CI, 0.891-0.979). Five of the CCs comprised both Ery^R^ and susceptible isolates. Among the resistant isolates, those harboring different resistance genes shared the same CC. Also, isolates belonging to different serotypes or recovered from different clinical sources were clustered in the same CC.

## Discussion

*S. agalactiae* is recognized as a frequent colonizing agent in pregnant women and is an important cause of neonatal sepsis and meningitis. Nevertheless, in the past decade GBS has been increasingly associated with invasive disease in non-pregnant adults [1,2].

The aim of this study was to phenotypically and genotypically characterize 434 GBS isolates obtained from different regions of Brazil, a continental country, with a large ethnic, climatic, social and economic diversity. Most of the previous studies performed in Brazil were limited to isolates from the South and Southeast regions [10-15]. This is the first study in Brazil encompassing the characterization of GBS isolates from 15 different locations representative of the five Brazilian geographical regions. Therefore, although sampling each geographic region and checking for variations among them was not a primary objective of the present study, it seemed useful and informative to display our results according to the geographical region. Nevertheless, our data might not fully and accurately represent each one of the five geographical regions.

Serotype determination has been traditionally applied in epidemiological studies on GBS and is an important aid in the development of broadly protective vaccines containing capsular polysaccharides or polysaccharides conjugated to protein [5]. The serotype distribution varies geographically and studies performed in European countries, United States and in Latin America have shown that serotypes Ia, Ib, III or V are usually the most frequently found [13,34]. In this study, serotypes Ia, Ib, II and V were the most commonly detected, in frequencies varying according to the region. Serotypes VI to VIII were not found, and these serotypes are still rarely reported from other locations [9]. Our results are in agreement with previous data obtained in earlier Brazilian studies with isolates obtained from the South and Southeast regions [10,15,35], as well as with data published in several other countries [24,25].

Serotypes were found in similar frequencies among isolates obtained from colonization as well as infections (*p* = 0.1392), suggesting that the capsule, despite being an essential factor for the virulence in GBS is not the only bacterial determinant responsible for the pathogenicity. Other studies have described the predominance of serotypes III and V among cases of infection in neonates and in non-pregnant adults, respectively [25,34] while serotype Ia was found to predominate among GBS causing invasive infections in non-pregnant adults in Portugal [36]. In Brazil, previous studies showed that serotypes Ia, II, III and V were found in 68.2% of isolates predominantly from vaginal specimens of asymptomatic pregnant woman [14]. On the other hand, serotype IV was detected in 13.1% of the isolates from Curitiba city, Paraná state, Brazil, being most of them from infectious cases [13], and serotypes Ib (34.9%) and Ia (25.6%) were predominant among a cohort of HIV-infected pregnant women in Rio de Janeiro city [35].

The occurrence of a nontypeable isolates rate of 6.2% in this study is consistent with the previously reported data [13]. Nevertheless, it should be pointed out that serotype IX was not investigated in the present study due to the lack of specific antiserum and that the detection of a serotype IX GBS isolate has been recently reported in the South region of Brazil [37]. Therefore, it is possible that the group of NT isolates may include representatives of this serotype.

The uniform susceptibility of GBS to beta-lactam antibiotics detected in this study is consistent with previously published data on *S. agalactiae* isolates in Brazil [10-15,30], as well as in several regions in the world [8,25]. However, an extensive network national surveillance should be implemented since isolates with reduced susceptibility to penicillin have been described in various regions of the world [16-18]. Although susceptibility of GBS to beta-lactam antibiotics was observed in the present study, reduced susceptibility has been reported elsewhere, underscoring the importance of an ongoing surveillance to monitor emergence of increased penicillin MICs among GBS isolates.

The low rates of resistance to erythromycin (4.1%) and clindamycin (3.0%) found in this study, are similar to that obtained in other studies conducted in Brazil, and some other countries in Latin America [11,13,15,25]. On the other hand, higher resistance rates have been observed in studies conducted in Asia, Europe, United States and Canada [6,22,23]. No correlation was found among erythromycin resistance and serotype, as previously observed [13]. Nonetheless, the predominance of serotype V among isolates with MLS_B_ resistance has been reported [8]. Erythromycin-resistant isolates in the present study were obtained from colonization or non-invasive infections, as previously described [13,15]. These data suggest that the use of erythromycin and clindamycin as second-choice drugs in individuals with penicillin allergy should be accompanied by routine susceptibility testing to ensure proper therapy. If this is not possible, then treatment with vancomycin is recommended by CDC [19].

Among the Ery^R^ isolates, the *ermA* gene was the most frequent. Isolates presenting the cMLS_B_ phenotype were mainly associated with the *erm*B gene, while those with iMLS_B_ or M phenotypes harbored the *erm*A gene. Other authors have shown similar results [38,39]. The *mef*A gene was not found in this study, as previously described in Brazil [11,13], in contrast with the results obtained in other studies, where the *mef*A gene was detected in the isolates expressing the M phenotype [6,38]. The causes for the absence of resistance genes in six isolates with MLS_B_ phenotype might reside in the fact that a great variety of genetic mechanisms can be associated with a single resistance phenotype. Here, we investigated the presence of the genes most commonly reported among GBS strains, however those 6 isolates could harbor less frequent determinants, such as *erm*C, *erm*F, *erm*Q, *erm*T, *msr*A or *msr*D; all of them already described among streptococci [40]. Also, mutations in 23S rRNA or in the genes encoding ribossomal proteins can also be speculated as possible causes of erythromycin-resistance in such strains.

Erythromycin-resistant isolates showed a slightly higher homogeneity by PFGE when compared to the susceptible strains analyzed. Nevertheless, a considerable genetic diversity was observed among the 42 isolates typed by PFGE, which clustered Ery^R^ and susceptible isolates in the same CC, suggesting that resistance to erythromycin is not necessarily a clonal characteristic among GBS strains included in the present study. On the other hand, the majority (64.7%) of the 17 Eri^R^ isolates were distributed among five of the nine CCs detected, suggesting that multiple clusters may have arisen simultaneously, mostly by independent acquisition of resistance genes by different strains. Similar results were previously shown in other studies [13,15,41]. Likewise, serotype does not seem to have a correlation with the classification of a GBS isolate in a clonal group. Relationships between the serotype and the source of isolation or between the serotypes and PFGE profiles were also not detected in another Brazilian study [14]. This fact could be explained by the occurrence of capsule switching between GBS isolates by horizontal gene transfer, as a result of pressures by immune response of the host [7]. One Ery^R^ isolate could not be typed by PFGE due to resistance to *Sma*I restriction. This phenomenon is not rare, and has been reported by other authors from different parts of the world [8,42,43]. Nevertheless, PFGE still represents an important molecular typing tool for GBS strains in Brazil, where, to our knowledge, other molecular methods such as MLST and MLVA have not or have rarely [37] been employed.

Resistance to tetracycline was detected in most of the isolates of this study, and the *tet*M gene was widely disseminated as previously reported in Brazil as well as in other countries [6,8,11,15,25]. All the isolates resistant to macrolides independent from the phenotypic and genotypic mechanism of resistance also had the *tet*M gene.

## Conclusions

The present study provides an overview of the distribution of serotypes and on antimicrobial susceptibility of GBS isolates from Brazil, the largest country in Latin America. The findings suggest that GBS isolates circulating in Brazil have a wide phenotypic and genotypic diversity and that macrolide-resistant isolates may arise by both clonal spread and independent acquisition of resistance genes. These data are important to help in designing prevention and treatment strategies for GBS infections in the region.

## Competing interests

The authors declare that they have no competing interests.

## Authors’ contributions

VGD, LMT and SELF conceived the study, participated in the design and coordination, collaborated in the capsular typing, performed the antimicrobial susceptibility testing and drafted the manuscript. VMNA, ANO, CAGD, AFAB, ELTA, MABS, RS, PCSR, CFF, MA, KM, AAA, LFP, CM, EFCM, PCFJ, MSGR, PSM, RBGB, JKP, LMDC participated in data collection and presumptive identification of isolates obtained in 5 Brazilian regions. TCAP performed the PFGE analysis and the PCR reactions. ACNB participated in the capsular typing, performed the antimicrobial susceptibility testing, performed the PCR reactions and maintained GBS isolate collections. All authors read and approved the final manuscript.

## Pre-publication history

The pre-publication history for this paper can be accessed here:

http://www.biomedcentral.com/1471-2334/14/323/prepub
